# Body shape concerns and behavioral intentions on eating disorders: a cross-sectional study of Chinese female university students using an extended theory of reasoned action model

**DOI:** 10.3389/fnut.2024.1501536

**Published:** 2024-12-24

**Authors:** Jingyi Zhao, Jing Zhao, Han Yuan, Zeng Gao

**Affiliations:** ^1^Physical Education Department, Nanjing Institute of Technology, Nanjing, China; ^2^College of Educational Science, Guangdong Preschool Normal College in Maoming, Maoming, China; ^3^Department of Physical Education, Kyungpook National University, Daegu, Republic of Korea; ^4^Department of Physical Education, Xiangtan University, Xiangtan, China; ^5^School of Educational Studies, Universiti Sains Malaysia, George Town, Pinang, Malaysia

**Keywords:** body shape concern, behavioral intention, eating disorders, female university students, theory of reasoned action

## Abstract

**Background:**

Weight and body shape concerns have become increasingly common among adolescents. Chinese university students show a high risk of eating disorder behaviors. This study aims to analyze the moderating effect of BMI on the relationships between body shape, attitudes, subjective norms, and eating disorder behavioral intentions among Chinese female university students using the Theory of Reasoned Action (TRA) model.

**Methods:**

A stratified random sample of 679 female Chinese university students (age, mean ± SD = 19.792 ± 1.007) participated in the study. The surveys comprised the Theory of Reasoned Action Questionnaire (TRA-Q) and the Body Shape Questionnaire (BS-Q) to assess their body shape concerns and behavioral intentions regarding eating disorders. Structural equation modeling was used to test the extended TRA model, with body shape as an additional predictor and BMI as a moderator.

**Results:**

Body shape positively affected attitudes (*β* = 0.444, *p* < 0.001), subjective norms (*β* = 0.506, *p* < 0.001), and intentions (*β* = 0.374, *p* < 0.001). BMI significantly moderated the relationships between attitudes (*t* = −3.012, *p* < 0.01), subjective norms (*t* = −2.678, *p* < 0.01), and body shapes (*t* = −4.485, *p* < 0.001) toward eating disorder intentions.

**Conclusion:**

Body shape and BMI directly influence eating disorder behavioral intentions among Chinese female university students. The findings suggest that young Chinese women’s eating disorder intentions are increasingly influenced by external factors related to body shape and BMI.

## 1 Introduction

Weight and body shape concerns (BSC) are increasingly prevalent among adolescents, contributing to significant physical and mental health challenges. These concerns often lead to psychological disorders such as depression, anxiety, paranoia, and eating disorders such as anorexia nervosa (AN), while also potentially contributing to obesity as a separate health condition (1–4). Body shape dissatisfaction can further result in obesity or malnutrition, negatively impacting self-esteem and leading to distressing conditions like social anxiety, phobias, and severe emotional disturbances (5, 6). Recent studies underscore the alarming prevalence of these issues among Chinese female university students. A survey of 2,023 participants found that 73.36% had attempted to lose weight, 30.55% were already underweight, and 57.39% desired to be thinner-indicating a rising trend of malnourished individuals in this demographic (7, 47). The high prevalence of eating disorders within this group is particularly concerning (8).

Media representation, family dynamics, and peer influences play critical roles in shaping female body image dissatisfaction, often leading to fears of negative judgment based on weight and BSC (9, 10). In severe cases, these pressures can lead to eating disorders such as bulimia (11). Across several Asian countries, including China, Japan, Korea, Taiwan, and Pakistan, high levels of body dissatisfaction and unhealthy eating attitudes have been documented, with gender being a significant factor (12). Moreover, Subjective norm (SN) and behavioral intention (BI) profoundly influence perceptions of BSC, especially among females, who are more likely to develop unhealthy eating behaviors in response to these pressures (13, 14). Previous research has shown that BSC can influence how individuals perceive and respond to social norms regarding eating behaviors (15). This relationship is particularly relevant in Chinese culture, where social pressure regarding body image can be intensified by personal BSC (16). The TRA suggests that an individual’s BIs are shaped by their Attitudes (AT) toward the behavior and the perceived social pressures, or SN, to perform or avoid that behavior. The TRA model has been extensively used to predict human BIs in various contexts (17, 18). For instance, studies have shown that AT and social influences strongly drive environmental practices (3), job-related attitudes and intentions (19), and mobile banking adoption (20).

Young female university students, particularly those who are obese, are highly susceptible to external influences, including media portrayals and societal standards, which contribute to body dissatisfaction and the development of eating disorders (6, 21). In China, the situation is increasingly concerning. Obesity rates among university students are rising, and many young women are placing greater importance on their image, often striving for an unhealthy thin ideal. This preoccupation with BSC, coupled with external influences such as media portrayals and peer pressure, significantly increases the risk of developing eating disorders (22, 48). Body mass index (BMI) plays a critical role in this context, influencing both the perception of body image and the health risks associated with obesity, including cardiovascular disease (47, 23). Previous research, including our cross-sectional survey, has demonstrated a significant positive correlation between BSC and eating disorder behaviors among Chinese university students, with gender acting as a moderating factor. Female students, in particular, are more vulnerable to external pressures that can lead to the development of eating disorders (7).

Building on this foundation, the study extends the traditional TRA model by incorporating BSC as an additional predictor variable and BMI as a moderating variable, to understand better BSC influences AT, SN, and BI related to eating disorders among Chinese female university students. This extension allows us to examine how BSC directly influence AT, SN, and BI related to eating disorders.

The research objectives are summarized as follows:

1. To identify the relationships between body shape concerns and attitudes, subjective norms, and behavioral intentions on eating disorders.
2. To analyze the moderating effect of BMI on these relationships among Chinese female university students.

## 2 Conceptual framework and hypotheses

### 2.1 Conceptual framework

The TRA model, developed by Feishbein and Ajzen (17), suggests that an individual’s BIs are influenced by their ATs and SNs. This model posits that certain beliefs and information shape BIs through the mediating effects of personal ATs and perceived social pressure (24, 25). The TRA model has been extensively applied across diverse studies, such as exploring the impact of self-esteem and body dissatisfaction on clothing behaviors among Generation men (26), assessing healthy eating habits (27), examining eating decisions among female university dieters and non-dieters (28), analyzing muscle endurance and BMI among university students (29), and understanding online purchasing intentions for exercise apparel among overweight and obese adults (30).

**Figure 1:**
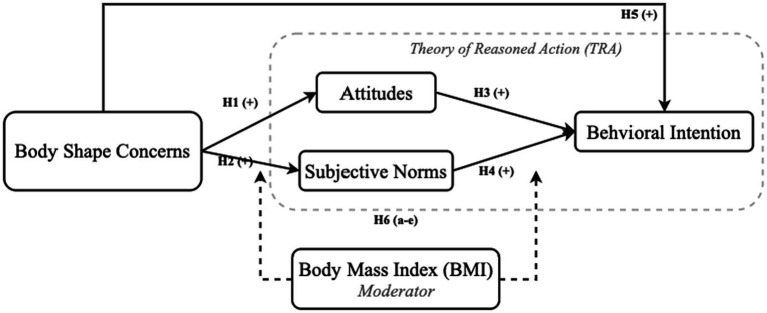
Conceptual framework.

Despite its widespread application, there has been limited research on the applicability of the TRA model to BSC and their influence on eating disorder BIs. This study addresses this gap by extending the TRA model to analyze the moderating effects of BMI on the relationships between BSC, AT, SN, and BI among Chinese female university students. The proposed research design integrates BSC into the extended TRA model to better understand how variations in BSC impact these relationships (see Figure 1).

### 2.2 Hypotheses

The research hypotheses are as follows:

*H1*: Body shape concerns positively affects attitudes.

*H2*: Body shape concerns positively affects subjective norms.

*H3*: Attitudes positively affect behavioral intentions.

*H4*: Subjective norms positively affect behavioral intentions.

*H5*: Body shape concerns positively affects behavioral intentions.

*H6 (a–e)*: BMI had a significant effect as a moderator variable on the relationship between body shape concerns, attitude, subjective norms, and behavioral intentions.

## 3 Methods

### 3.1 Participants

A random sample of 687 female university students was collected from April 20th to May 20th, 2024, at the Nanjing Institute of Technology. The participants’ ages as a continuous variable from 18 to 24 years (*M* = 19.792, SD = 1.007). After excluding 8 incomplete responses (1.16%), a final sample of 679 participants was analyzed. Data collection was conducted online from January 1st to April 1st, 2024. The participants’ ages were determined from questionnaire responses. Using G*Power 3.1 software, the required sample size was calculated to be 541, with an effect size (f^2^) of 0.05, an alpha error probability (*α*) of 0.05, and a power (1-*β*) of 0.99. This aligns with similar studies, such as Yang et al. (31) and Dubey and Sahu (32), which used comparable sample sizes. The study was approved by the university ethics committee. All participants were fully informed of the purpose, process, and potential risks of the study before participation and signed an informed consent form. Data collection was conducted after obtaining written consent from the participants.

### 3.2 Measures

#### 3.2.1 The TRA-Q

The TRA-Q has been used to prove a validated tool and used for evaluating BI on eating disorders (33). It consists of 23 items subdivided into 3 subscales attitudes (5 items), subjective norms (8 items), and behavioral intention (10 items) (33, 34). Participants used a 7-point Likert scale from 1 to 7, which were 1 (Strongly Disagree), 2 (Disagree), 3 (Somewhat Disagree), 4 (Neutral), 5 (Somewhat Agree), 6 (Agree), and 7 (Strongly Agree). The study adopted the Chinese version of the Theory of Reasoned Action Questionnaire (TRA-Q) parts of the theory of planned behavior model (33, 34). This tool has high criterion-related validity and internal consistency (*α* = 0.93) validated for the Chinese population (33, 34).

#### 3.2.2 The BS-Q

The BS-Q has been used to prove a validated tool and used for evaluating BSC (35). It consists of 8 items, and the questionnaire adopted a 6-point Likert scale from 1 to 6 (Rarely, Sometimes, Often, Usually, and Always) (8, 35). Its scores range from 8 to 48, and a score less than 19 indicates no concern with shape, 19 to 25 indicates mild concern with shape, 26 to 33 indicates moderate concern with shape, and over 33 indicates marked concern with shape (35). The study adopted a Chinese version of the Body Shape Questionnaire (BS-Q) (8, 35). The questionnaire demonstrated high reliability, with a coefficient of 0.94, and it has been validated for the Chinese population (7, 8, 35, 36).

### 3.3 Statistical analysis

The data were analyzed using SPSS version 24.0 statistical software, incorporating descriptive statistics, Pearson correlation analysis, path analysis, and moderation analysis. Structural equation modeling was conducted using SPSS PROCESS, employing maximum likelihood estimation. Model fit was assessed using multiple indices including CFI, TLI, RMSEA, and SRMR. The measurement model was evaluated before testing the structural model, following standard two-step structural equation modeling procedures. Descriptive statistics were used to calculate frequencies, percentages, and chi-squared tests for categorical variables, as well as t-tests for continuous variables. Pearson correlation analysis examined the relationships between all variables. Path analysis was employed to test hypotheses 1 through 5, while moderation analysis assessed the moderating role of BMI in the relationships between body shape concerns, attitudes, subjective norms, and behavioral intentions (hypothesis 6). All questionnaires used were validated Chinese versions from previous studies, ensuring consistency and cultural relevance in data collection.

## 4 Results

### 4.1 Demographic characteristics

This study involved 679 female university students from the Nanjing Institute of Technology in Nanjing, China. The average age of the participants was 19.792 years (SD = 1.007), with ages ranging from 18 to 24 years. Most students had a normal BMI (*n* = 493, 72.61%), while 82 students (12.08%) were classified as overweight. Three students (0.44%) were identified as obese, and 101 students (14.87%) were considered underweight. Additional demographic details are provided in Table 1.

**Table 1:** Demographic characteristics.

Items	Categories	All Participants (*n*)	%
Age	18 yrs.	55	8.10
	19 yrs.	186	27.39
	20 yrs.	298	43.89
	21 yrs.	107	15.76
	22 yrs.	23	3.39
	23 yrs.	7	1.03
	24 yrs.	3	0.44
Average age		19.792 ± 1.007	
BMI	Underweight	101	14.87
	Normal	493	72.61
	Overweight	82	12.08
	Obesity	3	0.44
Parents’ educational status	Elementary School	129	19.00
	Secondary School	242	35.64
	High School	156	22.97
	Bachelor and above	152	22.39
Family income	Low	307	45.21
	Average	316	46.54
	Good	56	8.25
Parents’ marital status	Married	626	92.19
	Divorced/Widowed	53	7.81

BMI is body mass index; Underweight is < 18.5 kg/m^2^; Normal is 18.5–24.9 kg/m^2^; Overweight is 25.0–29.9 kg/m^2^; Obesity is > 29.9 kg/m^2^; Low is below 16,443 RMB/year; Average is between 16,443 and 41,172 RMB/year; Good is above 41,172 RMB/year; ** *p* < 0.01.

### 4.2 Descriptive statistics

Table 2 shows demographic characteristics and descriptive statistics. The chi-squared test will be used to analyze four groups of BSC categorical variables, purposing further to confirm whether has a statistically significant difference in the demographic characteristics. Its result reported that BMI (*χ*2 = 72.548, *p* = 0.001 < 0.01) and parents’ educational status (*χ*^2^ = 35.917, *p* = 0.001 < 0.01) with BSC showed significant differences (*p* < 0.05). Other’s ages, family incomes, and parent’s marital status did not significantly difference (*p* > 0.05). All participants of average age with no concern with shape (NO-CS), mild concern with shape (MI-CS), moderate concern with shape (MO-CS), and marked concern with shape (MA-CS) respectively are 19.838 yrs. (1.054), 19.866 yrs. (1.012), 19.838 yrs. (1.015), and 19.882 yrs. (0.907). Due to differences in BMI and parents’ educational status, we divided the BMI into different level groups, namely underweight (< 18.5 kg/m^2^), normal (18.5–24.9 kg/m^2^), overweight (25.0–29.9 kg/m^2^), and obesity (>29.9 kg/m^2^), and the parents’ educational status into different level groups, namely elementary school, secondary school, high school, and bachelor and above. According to the significant difference analyses, we further determine if age, BMI, parents’ educational status, family income, and parent’s marital status had significant differences from all variables. We used the different groups of BMI as continuous variables for analysis.

**Table 2:** Descriptive statistics of the BS-Q scores.

Categories	Body shape questionnaire				*χ^2^*	*p*
	NO-CS *n* (%)	MI-CS *n* (%)	MO-CS *n* (%)	MA-CS *n* (%)		
Age						
18 yrs.	14 (7.87)	21 (8.50)	17 (9.14)	3 (4.41)	19.725	0.348
19 yrs.	53 (29.78)	66 (26.72)	47 (25.27)	20 (29.41)		
20 yrs.	80 (44.94)	113 (45.75)	75 (40.32)	30 (44.12)		
21 yrs.	22 (12.36)	33 (13.36)	40 (21.51)	12 (17.65)		
22 yrs.	5 (2.81)	10 (4.05)	5 (2.69)	3 (4.41)		
23 yrs.	4 (2.25)	1 (0.40)	2 (1.08)	0		
24 yrs.	0	3 (1.21)	0	0		
Average Age	19.838 ± 1.054	19.866 ± 1.012	19.838 ± 1.015	19.882 ± 0.907	–	–
BMI						
Underweight	51 (28.65)	32 (12.96)	10 (5.38)	8 (11.76)	72.548	0.001^**^
Normal	113 (63.48)	195 (78.95)	145 (77.96)	40 (58.82)		
Overweight	14 (7.87)	20 (8.10)	30(16.13)	18 (26.47)		
Obesity	0	0	1(0.54)	2 (2.94)		
Parents’ educational status						
Elementary School	36 (20.22)	55 (22.27)	30 (16.13)	8 (11.76)	35.917	0.001^**^
Secondary School	51 (28.65)	114 (46.15)	59 (31.72)	18 (26.47)		
High School	44 (24.72)	38 (15.38)	54 (29.03)	20 (29.41)		
Bachelor and above	47 (26.40)	40 (16.19)	43 (23.12)	22 (32.35)		
Family income						
Low	82 (46.07)	97 (39.27)	94 (50.54)	34 (50.00)	8.760	0.188
Average	86 (48.31)	124 (50.20)	78 (41.94)	28 (41.18)		
Good	10 (5.62)	26 (10.53)	14 (7.53)	6 (8.82)		
Parents’ marital status						
Married	169 (94.94)	228 (92.31)	164 (88.17)	65 (95.59)	7.144	0.067
Divorced/Widowed	9 (5.06)	19 (7.69)	22 (11.83)	3 (4.41)		

BMI is body mass index; NO-CS is no concern with shape; MI-CS is mild concern with shape; MO-CS is moderate concern with shape; MA-CS is marked concern with shape; Underweight is < 18.5 kg/m^2^; Normal is 18.5–24.9 kg/m^2^; Overweight is 25.0–29.9 kg/m^2^; Obesity is > 29.9 kg/m^2^; Low is below 16,443 RMB/year; Average is between 16,443 and 41,172 RMB/year; Good is above 41,172 RMB/year; **p < 0.01.

### 4.3 Reliability and validity

Table 3 demonstrates that all data underwent reliability and validity checks using factor loading, CR values, AVE values, Cronbach’s alpha, and KMO values. All items showed factor loadings above 0.6, CR values exceeding 0.80, AVE values above 0.45, Cronbach’s alpha values over 0.80, and KMO values surpassing 0.70. The AVE values being greater than 0.45 indicate suitable convergent validity, meaning that each construct’s measurement items can explain more than 45% of the total variance. Although AVE values ideally should be above 0.50 (37), AVE values above 0.45 are acceptable when the CR value exceeds 0.80 (38, 39). This suggests high consistency and shared variance among the construct’s measurement items. Consequently, the results indicate that all questionnaire items and structures are reliable and valid for Chinese female university students.

**Table 3:** Reliability and validity.

Variables	Items	Factor loading	CR	AVE	Cronbach α	KMO
BSC	BS1	0.711	0.935	0.643	0.934	0.922
	BS2	0.804				
	BS3	0.854				
	BS4	0.833				
	BS5	0.871				
	BS6	0.851				
	BS7	0.853				
	BS8	0.844				
AT	AT1	0.796	0.830	0.494	0.829	0.788
	AT2	0.777				
	AT3	0.765				
	AT4	0.800				
	AT5	0.715				
SN	SN1	0.618	0.871	0.461	0.868	0.879
	SN2	0.795				
	SN3	0.687				
	SN4	0.734				
	SN5	0.778				
	SN6	0.655				
	SN7	0.825				
	SN8	0.676				
BI	BI1	0.744	0.897	0.466	0.894	0.849
	BI2	0.749				
	BI3	0.781				
	BI4	0.695				
	BI5	0.776				
	BI6	0.637				
	BI7	0.673				
	BI8	0.770				
	BI9	0.656				
	BI10	0.702				

*χ2 = 3647.704; df = 428; PGFI = 0.605; CR is construct reliability; AVE is average variance extracted; KMO is Kaiser-Meyer-Olkin.*

Table 4 shows the discriminant validity and correlations. The AVE square root value of BSC is greater than the maximum absolute value of the inter-factor correlation coefficient (0.802 > 0.590), indicating that BSC has good discriminant validity. The AVE square root value of AT is greater than the maximum absolute value of the inter-factor correlation coefficient (0.703 > 0.475), indicating that AT has good discriminant validity. The AVE square root value of SN is greater than the maximum absolute value of the inter-factor correlation coefficient (0.679 > 0.591), indicating that SN has good discriminant validity. The AVE square root value of BI is greater than the maximum absolute value of the inter-factor correlation coefficient (0.683 > 0.591), indicating that BI also has good discriminant validity.

**Table 4:** Discriminant validity and correlations.

Variables	1	2	3	4
1.BS	**0.802**			
2. AT	0.444	**0.703**		
3.SN	0.506	0.475	**0.679**	
4. BI	0.590	0.410	0.591	**0.683**

Diagonal bold is the average variance extracted (AVE); the diagonal bottom is the square value of the correlation coefficient.

### 4.4 Correlation of all variables

Table 5 reports the Pearson correlation coefficients. The results that BI with AT (*r* = 0.410, *p* = 0.001 < 0.05), SN (*r* = 0.591, *p* = 0.001 < 0.05), BMI (*r* = 0.129, *p* = 0.001 < 0.05), and parents’ educational status (*r* = 0.129, *p* = 0.003 < 0.05) were all significant, indicating that BI with AT, SN, BMI, and parents’ educational status were positively correlated. BI with age (*r* = 0.039, *p* = 0.308 > 0.05), family Income (*r* = −0.006, *p* = 0.872 > 0.05), and parents’ marital status (*r* = 0.058, *p* = 0.129 > 0.05) were not significant, indicating that they were not correlated. BSC with AT (*r* = 0.444, *p* = 0.001 < 0.05), SN (*r* = 0.506, *p* = 0.001 < 0.05), IN (*r* = 0.590, *p* = 0.001 < 0.05), and BMI (*r* = 0.247, *p* = 0.001 < 0.05) were all significant, indicating that BSC was positively correlated with AT, SN, BI, and BMI. In addition, BSC with age (*r* = 0.031, *p* = 0.417 > 0.05), parents’ educational status (*r* = 0.060, *p* = 0.119 > 0.05), family Income (*r* = −0.005, *p* = 0.900 > 0.05), and parents’ marital status (*r* = 0.039, *p* = 0.311 > 0.05) did not show significance, indicating that they were not correlated.

**Table 5:** Pearson correlation analysis.

Variables		1	2	3	4	5	6	7	8	9
1. BSC	*r*	1								
	*p*	–								
2. AT	*r*	**0.444** ^**^	1							
	*p*	0.001	–							
3. SN	*r*	**0.506** ^**^	**0.475** ^**^	1						
	*p*	0.001	0.001	–						
4. BI	*r*	**0.590** ^**^	**0.410** ^**^	**0.591** ^**^	1					
	*p*	0.001	0.001	0.001	–					
5. Age	*r*	0.031	**−0.112** ^**^	−0.056	0.039	1				
	*p*	0.417	0.004	0.144	0.308	–				
6. BMI	*r*	**0.247** ^**^	**0.089** ^*^	**0.121** ^**^	**0.129** ^**^	**−0.084** ^*^	1			
	*p*	0.001	0.020	0.002	0.001	0.028	–			
7. Parents’ educational status	*r*	0.060	**0.085** ^*^	0.061	**0.129** ^**^	**−0.184** ^**^	0.043	1		
	*p*	0.119	0.026	0.109	0.003	0.001	0.260	–		
8. Family income	*r*	−0.005	0.001	**−0.102** ^**^	−0.006	−0.045	0.075	−0.028	1	
	*p*	0.900	0.976	0.008	0.872	0.239	0.051	0.460	–	
9. Parents’ marital status	*r*	0.039	0.044	0.101	0.058	**−0.148** ^**^	0.031	0.043	**−0.169** ^**^	1
	*p*	0.311	0.256	**0.009** ^**^	0.129	0.001	0.421	0.261	0.001	–

** *p* < 0.01.

### 4.5 Hypotheses result from H1 to H5

Table 6 presents the hypotheses of the results through the Path analysis, revealing that BSC positively affect ATs (*β* = 0.444, CR = 12.914, *p* = 0.001 < 0.01) and SNs (*β* = 0.506, CR = 15.273, *p* = 0.001 < 0.01) among Chinese female university students, thus the Hypotheses 1 and Hypotheses 2 results were supported. Moreover, ATs (*β* = 0.070, CR = 2.225, *p* = 0.026 < 0.05), SNs (*β* = 0.372, CR = 11.378, *p* = 0.001 < 0.01), and BSC (*β* = 0.374, CR = 10.526, *p* = 0.001 < 0.01) all positively affect BI among Chinese female university students, so the Hypotheses 3, Hypotheses 4, and Hypotheses 5 results also were supported.

**Table 6:** Path analysis.

Hypothesis	Relationship			*Path Coeff.*	S.E.	*CR*	*p*	Results
	*X*	→	*Y*					
H1	BSC	→	AT	0.444	0.040	12.914	0.001^**^	Supported
H2	BSC	→	SN	0.506	0.031	15.273	0.001^**^	Supported
H3	AT	→	BI	0.070	0.027	2.225	0.026^*^	Supported
H4	SN	→	BI	0.372	0.034	11.378	0.001^**^	Supported
H5	BSC	→	BI	0.374	0.034	10.526	0.001^**^	Supported

*p < 0.05; **p < 0.01.

### 4.6 Hypothesis result of H6

Table 7 shows different BMI groups as a moderation analysis between BSC, AT, SN, and BI. Different BMI groups had no significant effect as a moderator variable on the relationship between BSC toward AT (*t* = −1.318, *p* = 0.188 > 0.05), and SN (*t* = −1.838, *p* = 0.066 > 0.05), indirect that different BMI groups no effect. However, different BMI groups had a significant effect as a moderator variable on the relationship between AT (*t* = −3.012, *p* = 0.003 < 0.01), SN (*t* = −2.678, *p* = 0.008 < 0.01), and BSC (*t* = −4.485, *p* = 0.001 < 0.01) toward the BI. Therefore, different BMI groups have significant moderating effects from AT, SN, and BSC to BI.

**Table 7:** Moderation analysis.

Hypothesis	Relationship			BMI					Results
	*X*	→	*Y*	*B*	S.E.	*t-values*	*p*	*β*	
H6a	BSC	→	AT	−0.085	0.065	−1.318	0.188	−0.045	Not Supported
H6b	BSC	→	SN	−0.092	0.050	−1.838	0.066	−0.061	Not Supported
H6c	AT	→	IN	−1.143	0.047	−3.012	0.003^**^	−0.106	Supported
H6d	SN	→	IN	−0.142	0.053	−2.678	0.008^**^	−0.083	Supported
H6e	BSC	→	IN	−0.219	0.049	−4.485	0.001^**^	−0.138	Supported

*p < 0.05; **p < 0.01.

## 5 Discussion

This study investigated the relationship between BSCs and BI toward eating disorders among Chinese female university students using the extended TRA model, with a specific focus on the moderating role of BMI. The results reveal several key findings: (1) Impact of BSCs: BSCs positively influence ATs, SNs, and BIs related to eating disorders among Chinese female university students; (2) Role of ATs and SNs: Both ATs and SNs are positively associated with BIs in this demographic; (3) Moderating Effect of BMI: BMI significantly moderates the relationships between BSCs, ATs, SNs, and BIs. Additionally, the study found significant correlations between age, parents’ educational status, family income, and parents’ marital status with BSC, AT, SN, and BI.

These findings align with prior research indicating that BMI and BSCs are significant risk factors for eating disorders globally (2, 4, 7, 8, 11, 40). However, our findings also extend previous knowledge by emphasizing the moderating role of BMI in these relationships, highlighting nuanced interactions between BSCs and BIs. In China, the prevalence of eating disorder behaviors is notably high across different BMI categories, with detrimental effects on both physical and mental health (7, 8). These results corroborate earlier studies suggesting that BSCs increase the risk of eating disorders and associated conditions, such as cardiovascular disease (23) and mental health issues (3, 4, 11).

The study also supports the notion that high BSC is associated with various eating disorders, including binge eating disorder, bulimia, and anorexia (47, 21). For instance, individuals with low weight status driven by BSCs may develop anorexia (11, 47, 9, 10), while those in the obesity range may experience heightened appearance anxiety and social anxiety disorders (1, 3, 41, 42). Importantly, our study highlights the differential impacts of BS across BMI levels, reinforcing the need for tailored intervention strategies that consider BMI-specific dynamics in addressing disordered eating intentions.

The study underscores the interconnectedness of BSC, AT, SN, and BI regarding eating disorders. Individual differences were evident across BMI categories, parental educational levels, and family income groups of young female university students in China (7, 8). For instance, students from families with higher educational backgrounds showed different patterns of BSCs compared to those from less educated families (3, 8, 19). Similarly, family income levels appeared to moderate the relationship between BSCs and eating disorder intentions, though these effects varied considerably among individuals (8). However, the strength and nature of these relationships showed considerable individual variation, particularly across different BMI categories (7). Students with higher BMI demonstrated distinct patterns of BSCs and BIs compared to those with lower BMI, suggesting the need for tailored intervention approaches (7, 43).

Notably, this study addresses a gap in the literature, as there is limited research applying the extended TRA model to analyze BSC in predicting eating disorder BIs (17). Previous studies have primarily examined the general applicability of the TRA model, whereas our study provides a more targeted extension by incorporating BSC and BMI-specific moderating effects. Research has demonstrated that personal BIs significantly influence ATs, and that personal BIs and SNs are correlated (3, 19, 20, 44, 45). In China, the growing concern among young females about BSC is well-documented (47), and BMI exacerbates the thin ideal, impacting physical health (47). Conversely, high BMI or obesity is linked to an increased risk of cardiovascular disease (23) and mental health disorders (8, 46).

This study does have limitations. While our sample included students from various backgrounds, the homogeneity of the university setting may not fully capture the range of individual differences present in the broader population. The analysis of individual variations was limited by the focus on BMI as the primary moderating variable, potentially overlooking other important personal characteristics that could influence eating disorder behaviors. The reliance on self-reported data from Chinese female university students introduces potential bias and inaccuracies due to recall and self-reporting issues. Individual differences in self-perception and reporting accuracy could affect the reliability of the measurements. Future research should aim to expand the sample size and include a more diverse population to enhance representativeness and reliability. Moreover, integrating objective measures such as biomarkers or physiological data could provide more robust insights into the mechanisms driving these relationships. Despite these limitations, the study offers valuable insights into the relationship between BSC and eating disorder BIs, highlighting the need for targeted interventions. Future research should not only explore a broader range of individual differences and psychological factors such as media influences, self-esteem, personality traits, cultural background, and stress but also adopt longitudinal designs to uncover causal pathways and temporal dynamics of these associations.

## 6 Conclusion

The study underscores the interconnectedness of BSC, AT, SN, and BI regarding eating disorders. The findings highlight significant individual variations in these relationships, suggesting that one-size-fits-all approaches to eating disorder prevention may be insufficient. The findings contribute to the existing literature by highlighting the moderating role of BMI in these relationships, offering a more nuanced understanding of the factors influencing eating disorder intentions. The distinct impacts observed across different individual characteristics underscore the importance of personalized intervention strategies. Future research and clinical practice should consider these individual differences when developing prevention and treatment programs. For young female university students in China, BSC significantly influences their AT, SN, and BI. Furthermore, the distinct impacts of BMI underscore the importance of personalized intervention strategies tailored to individuals with varying BMI levels. Future research should explore additional variables and control factors to develop comprehensive prevention and treatment strategies, ultimately contributing to the improved mental and physical health of various demographic groups. By extending the TRA model to include BSC and BMI, this study provides a valuable framework for future investigations into eating disorders and related interventions, while emphasizing the need to account for individual variations in risk factors and treatment responses.

## Data Availability

The original contributions presented in the study are included in the article/supplementary material, further inquiries can be directed to the corresponding author.
